# Impact of Preoperative Diabetes Mellitus on Postoperative Outcomes in Elective Pancreatic Surgery and Its Implications for Prehabilitation Practice

**DOI:** 10.1097/MPA.0000000000002300

**Published:** 2024-01-26

**Authors:** Allard G. Wijma, Heleen Driessens, Maarten W. Nijkamp, Frederik J.H. Hoogwater, Peter R. van Dijk, Joost M. Klaase

**Affiliations:** From the ∗ Division of Hepato-Pancreato-Biliary Surgery and Liver Transplantation, Department of Surgery; † Division of Endocrinology, Department of Internal Medicine, University Medical Center Groningen, University of Groningen, Groningen, the Netherlands.

**Keywords:** glycemic control, pancreatoduodenectomy, perioperative care pathway, perioperative risk factors, prehabilitation

## Abstract

**Objectives:**

Patients with pancreatic disease(s) have a high risk of developing diabetes mellitus (DM). Diabetes mellitus is associated with adverse postoperative outcomes. This study aimed to investigate the prevalence and effects of DM on postoperative outcomes in pancreatic surgery.

**Methods:**

Subgroup analysis of a prospective cohort study conducted at an academic hospital. Patients undergoing pancreatoduodenectomy between January 2019 and November 2022 were included and screened for DM preoperatively using glycated hemoglobin (HbA1c). New-onset DM was diagnosed based on HbA1c ≥ 6.5% (48 mmol/mol). Postoperative outcomes were compared between patients with and without DM.

**Results:**

From 117 patients, 29 (24.8%) were given a diagnosis of DM, and of those, 5 (17.2%) were diagnosed with new-onset DM, and 15 (51.8%) displayed poorly controlled preoperative DM (HbA_1c_ ≥ 7% [53 mmol/mol]). The incidence of surgical site infections (48.3% vs 27.3% in the non-DM group; *P* = 0.04) was higher for patients with DM. This association remained significant after adjusting for confounders (odds ratio, 2.60 [95% confidence interval, 1.03–6.66]; *P* = 0.04).

**Conclusions:**

One-quarter of the patients scheduled for pancreatoduodenectomy had DM; over half of them had poor glycemic control. The association between DM status and surgical site infections revealed in this study emphasizes the importance of adequate preoperative glycemic control.

Diabetes mellitus (DM) is a leading cause of mortality and reduced life expectancy and is therefore considered one of the most significant global public health concerns.^1^ Alarmingly, the latest forecasting trends predict a fast rise in the prevalence of DM in the coming decades.^1,2^ Unhealthy lifestyles, such as sedentary lifestyles and unhealthy diets that lead to obesity and elevated fasting plasma glucose, are the predominant cause for the increase in the prevalence of DM.^2,3^ On account of this increase in prevalence, the number of patients with DM undergoing surgery will also increase.

In studies evaluating the effect of DM on postoperative outcomes in various surgical populations, DM was associated with increased postoperative morbidity, high surgical site infection (SSI) rates, and prolonged hospital stay.^4–8^ Furthermore, perioperative hyperglycemia in patients without DM was also associated with adverse postoperative outcomes, emphasizing the importance of normoglycemia in surgical patients.^9,10^ Diabetes mellitus and hyperglycemia are significant risk factors for postoperative complications, and adequate perioperative glycemic control should be prioritized to prevent adverse postoperative outcomes. The glycated hemoglobin (HbA_1c_) level is considered a suitable tool for diagnosing new-onset DM and monitoring long-term glycemic control because it reflects the average plasma glucose level over approximately 3 months.^11–13^

Despite substantial evidence linking DM to adverse postoperative outcomes in surgical patients, literature on DM in pancreatic surgery is scarce and inconclusive.^14–19^ Diabetes mellitus in patients undergoing pancreatic surgery poses unique challenges, which are not always recognized in everyday practice. Patients with pancreatic cancer are at an increased risk of developing DM because of a disease-related altered endocrine homeostasis caused by increased cytokine production and subsequent insulin resistance, also known as type 3c or pancreatogenic DM.^20,21^ As a result, DM is more prevalent in patients scheduled for pancreatic surgery than in the general population.^20^ Furthermore, because the resection of pancreatic parenchyma may result in endocrine pancreas insufficiency, new-onset DM is a common short- and long-term complication of pancreatic surgery.^20^

This study screened for DM using preoperative HbA_1c_ levels, revealing the prevalence of new-onset and poorly controlled DM in patients scheduled for pancreatic surgery. In addition, it provides insight into the effect of DM on SSIs and suggests implications for prehabilitation practice.

## MATERIALS AND METHODS

### Study Design and Setting

In this subgroup analysis of a single-center prospective cohort study,^22^ the preoperative prevalence of (new-onset) diabetes and its effect on postoperative outcomes were investigated in patients undergoing pancreatoduodenectomy (ie, pylorus-preserving pancreatoduodenectomy, pylorus-resecting pancreatoduodenectomy, or Whipple procedure) between January 2019 and November 2022 at the University Medical Center Groningen in the Netherlands. Patients were divided into a DM group and a non-DM group, with the former also including patients with new-onset DM. Patients with missing preoperative HbA_1c_ values and known type 1 DM were excluded from the analysis. Comparisons were made between (1) patients with and without DM and (2) patients with DM and adequate glycemic control and those with DM without adequate glycemic control. This study is part of the “Frail study” (Netherlands research registration number 201800293), in which a new preoperative care pathway was developed for patients scheduled to undergo hepato-pancreato-biliary surgery.^22^ All included patients completed the informed consent process, which was approved by the institutional review board of the University Medical Center Groningen. This study was performed in accordance with the ethical standards set by the Declaration of Helsinki.

### Assessment of DM

During the first outpatient clinic visit for each patient, a blood sample was drawn to determine the preoperative HbA_1c_ level. The diagnosis of existing DM was based on the patient's medical history and use of blood glucose–lowering medication. Preexisting DM was considered poorly controlled in patients with an HbA_1c_ ≥ 7% (53 mmol/mol).^11,13^ Moreover, patients with an HbA_1c_ ≥ 6.5% (48 mmol/mol) were given a diagnosis of new-onset DM.^13^ Patients with new-onset or poorly controlled DM were referred to their general practitioner for preoperative glycemic control support.

### Prehabilitation Outpatient Clinic

As part of the new preoperative care pathway, all patients scheduled to undergo hepato-pancreato-biliary surgery at the hospital were referred to the prehabilitation outpatient clinic for preoperative screening and assessment of potentially modifiable patient-related risk factors.^22^ In addition to screening for new-onset and poorly controlled DM, patients were screened for physical fitness and were advised to participate in an exercise program to improve their physical fitness before surgery if they were found to have low physical fitness. Moreover, a specialized dietician screened patients for malnutrition and provided them with dietary advice and/or nutritional supplements and pancreatic enzyme replacement therapy. Afterward, patients were screened for anxiety and depression symptoms and, if necessary, referred to a psychologist to enhance their mental resilience. Preoperative anemia was investigated by measuring hemoglobin level and iron status, and appropriate treatment was administered to treat the underlying cause of anemia in patients. Patients' frailty was determined using 2 questionnaires: the Groningen Frailty Indicator and the Robinson Frailty Score.^23,24^ Patients who were considered frail were referred to a geriatrician for a comprehensive geriatric assessment to implement a proactive integrated care plan for the postoperative period. Finally, substance abuse (ie, tobacco use and alcohol consumption) was assessed. Patients were strongly advised to stop substance abuse, and appropriate professional support was offered. The aim of this multimodal prehabilitation program was to identify patient-related risk factors, allowing for a personalized approach to optimize these risk factors before surgery.

### Data Collection and Study End Points

Demographic data, intraoperative and pathology details, and 30-day postoperative outcomes were collected from patient charts. The risk of postoperative pancreatic fistula was calculated using the Dutch Pancreatic Cancer Group fistula risk score for pancreatoduodenectomy.^25^ Complications specific to pancreatic surgery were recorded and graded according to the definitions of the International Study Group of Pancreatic Surgery.^26^ Moreover, postoperative complication severity was graded using the Clavien-Dindo classification system.^27^ Red blood cell (RBC) transfusion was defined as any allogeneic RBC transfusion in the perioperative period. Finally, hospital readmissions were defined as any unplanned readmission within 30 days of the initial discharge. This study's primary end point was the risk of developing an SSI, defined as any infection affecting the incision, deep tissue, or organ space. Secondary end points included the length of hospital stay in days, calculated from the day of surgery until the day of discharge. Because the length of stay was not normally distributed, it was categorized based on the median value of the total cohort and then used for regression analysis. Other secondary end points included postoperative complications, RBC transfusions, in-hospital mortality, and hospital readmissions.

### Statistical Analysis

The normality of continuous data was assessed using the Shapiro-Wilk test and QQ plots. Continuous variables were presented as mean with SD or as median with interquartile range based on normality of distribution. Categorical data were presented as numbers and percentages. The differences between groups (ie, between [1] patients with and without DM and [2] patients with DM and adequate glycemic control and those with DM without adequate glycemic control) were calculated using Student *t* test, Mann-Whitney *U* test, χ^2^ test, or Fisher exact test, as appropriate. In addition, univariable and multivariable logistic regression analyses were performed to assess the effect of DM on relevant postoperative outcome variables. To avoid collinearity, only a limited number of response variables considered clinically related to the outcome variables (ie, preoperative HbA_1c_, age, body mass index [BMI], estimated intraoperative blood loss, and operating time) were used in the regression models. All models yielded an estimated regression coefficient (β) and odds ratio with a 95% confidence interval (CI). To test for effect modification by BMI, an interaction term (ie, study cohort × BMI) was included. The R software version 4.2.2 (R Foundation for Statistical Computing, Vienna, Austria) was used for statistical analysis, particularly the “tidyverse” and “ggplot2” packages. In all analyses, a *P* value < 0.05 was considered statistically significant.

## RESULTS

### Patient Characteristics

**FIGURE 1:**
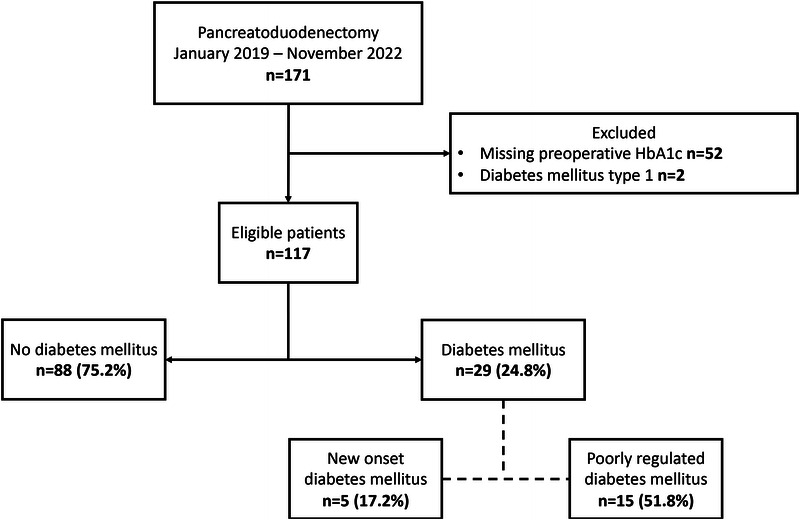
Overview of diabetes mellitus in patients undergoing pancreatoduodenectomy.

Between January 2019 and November 2022, 171 patients underwent a pancreatoduodenectomy. In 117 of these patients, HbA_1c_ was preoperatively assessed (Fig. 1). Of the included patients, 29 (24.8%) were determined to have DM, of whom 5 (17.2%) were given a diagnosis of new-onset DM (with a mean age of 64 years, a female sex ratio of 60%, and a median HbA_1c_ of 57 mmol/mol). Furthermore, DM was considered poorly controlled in 15 (51.8%) of the patients with DM, with HbA_1c_ levels ≥ 7% (53 mmol/mol). The median diabetes duration was 9 versus 11.5 years in the well-controlled and poorly controlled DM patient groups, respectively.

Table 1 presents an overview of the baseline patient characteristics of the study groups. In both the DM and non-DM groups, the mean age and female sex ratio were similar. As predicted, patients in the DM group had higher HbA_1c_ levels than those in the non-DM group. Moreover, the mean BMI was higher in the DM group, and the presence of more comorbidities resulted in more patients in the DM group having a Charlson Comorbidity Index ≥ 4 and American Society of Anesthesiologists classification ≥ 3.

**TABLE 1:** Baseline Patient Characteristics

	Non-DM n = 88 (75.2%)	DM n = 29 (24.8%)	*P*
Mean age, y	65.5 ± 9.3	67.5 ± 8.1	0.31
Female sex, n (%)	46 (52.3)	13 (44.8)	0.49
Mean BMI, kg/m^2^	25.3 ± 4.7	27.5 ± 4.8	**0.03**
Charlson Comorbidity Index ≥ 4, n (%)	30 (34.1)	19 (65.5)	**0.003**
ASA classification ≥ 3, n (%)	25 (28.4)	17 (58.6)	**0.003**
Median HbA_1c_, mmol/mol	37 (34–41)	54 (46–58)	**<0.001**
Comorbidities, n (%)			
Hypertension	28 (31.8)	23 (79.3)	**<0.001**
Cardiac	8 (9.1)	7 (24.1)	**0.04**
Pulmonary	13 (14.8)	4 (13.8)	1.00
Substance abuse, n (%)			
Tobacco	23 (26.1)	6 (20.7)	0.56
Alcohol	42 (47.7)	15 (51.7)	0.71
Neoadjuvant chemotherapy, n (%)	8 (9.1)	5 (17.2)	0.30

Data are presented as mean ± standard deviation, median (interquartile range), or number (%).

Significant values are indicated in bolded numbers.

ASA indicates American Society of Anesthesiologists; BMI, body mass index; DM, diabetes mellitus; HbA_1c_, glycated hemoglobin.

### Intraoperative Characteristics and Postoperative Outcomes

No differences were observed in intraoperative and pathology details between groups (Table 2). The operating time and estimated intraoperative blood loss was somewhat higher in the DM group, although for both variables, this did not reach statistical significance. Concerning the postoperative course, the DM group had a general tendency toward poor postoperative outcomes (Table 3). The median length of hospital stay was 1.5 days longer for patients in the DM group (13.5 vs 12 days in the DM and non-DM groups, respectively), despite lacking statistical significance. Although the incidence of severe complications, defined as a Clavien-Dindo grade ≥ 3b, was higher in the DM group (20.7% vs 9.1% in the DM and non-DM groups, respectively; *P* = 0.10), the rate of complications specific to pancreatic surgery was similar between groups. The number of patients requiring postoperative RBC transfusion was higher in the DM group; nevertheless, this effect did not reach statistical significance (31% vs 22.7% in the DM and non-DM groups, respectively; *P* = 0.37). The risk of SSI, the primary end point of this study, was significantly higher in the DM group (48.3% vs 27.3% in the DM and non-DM groups, respectively; *P* = 0.04). Finally, the rate of hospital readmissions was slightly higher in the DM group (20.7% vs 12.5% in the DM and non-DM groups, respectively; *P* = 0.26) but did not differ significantly.

**TABLE 2:** Intraoperative and Pathology Details

	Non-DM n = 88 (75.2%)	DM n = 29 (24.8%)	*P*
Intraoperative details			
Complementary resection, n (%)	5 (5.7)	2 (6.9)	1.00
Vascular resection, n (%)			
Arterial	4 (4.5)	2 (6.9)	0.64
Venous	20 (22.7)	10 (34.5)	0.21
Operating time, min	474 (413–534)	512 (469–569)	0.13
Estimated intraoperative blood loss, mL	500 (300–731)	550 (400–950)	0.16
Calculated POPF risk,* n (%)			0.87
Low	15 (17.1)	6 (20.7)	
Intermediate	56 (63.6)	17 (58.6)	
High	17 (19.3)	6 (20.7)	
Pathology details			
Origin of tumor, n (%)			0.85
Pancreas	43 (48.9)	16 (55.2)	
Distal CBD	18 (20.4)	6 (20.7)	
Ampulla of Vater	16 (18.2)	6 (20.7)	
Duodenum	8 (9.1)	1 (3.4)	
Other	3 (3.4)	—	
Histological diagnosis, n (%)			0.78
Adenocarcinoma	61 (69.3)	21 (72.3)	
Neuroendocrine	3 (3.4)	2 (6.9)	
IPMN	7 (8.0)	1 (3.5)	
Intestinal adenoma	6 (6.8)	1 (3.5)	
Other	11 (12.5)	4 (13.8)	

Data are presented as number (%) or median (interquartile range).

CBD indicates common bile duct; DM, diabetes mellitus; IPMN, intraductal papillary mucinous neoplasm; POPF, postoperative pancreatic fistula.

*Dutch Pancreatic Cancer Group fistula risk score for pancreatoduodenectomy.^25^

**TABLE 3:** Postoperative Outcomes

	Non-DM n = 88 (75.2%)	DM n = 29 (24.8%)	*P*
Length of hospital stay, d	12 (9–19.5)	13.5 (9–22)	0.70
Length of hospital stay ≥ 12 d	44 (50)	17 (58.6)	0.38
Surgery specific complications, n (%)		
POPF grade ≥ B	23 (26.1)	8 (27.6)	0.88
DGE grade ≥ B	23 (26.1)	9 (31)	0.61
BL grade ≥ B	3 (3.4)	—	0.57
PPH grade ≥ B	7 (8.0)	3 (10.4)	0.71
CL grade ≥ B	6 (6.8)	3 (10.4)	0.69
SSI, n (%)	24 (27.3)	14 (48.3)	**0.04**
Superficial incisional, n	18	10	
Deep incisional, n	6	2	
Organ space, n	—	2	
RBC transfusion, n (%)	20 (22.7)	9 (31)	0.37
Clavien-Dindo ≥ 3b complication, n (%)	8 (9.1)	6 (20.7)	0.10
In-hospital mortality, n (%)	2 (2.3)	1 (3.5)	1.00
Hospital readmission, n (%)	11 (12.5)	6 (20.7)	0.26

Data are presented as median (interquartile range) or number (%).

Significant values are indicated in bolded numbers.

BL indicates bile leakage; CL, chyle leakage; DGE, delayed gastric emptying; DM, diabetes mellitus; POPF, postoperative pancreatic fistula; PPH, postpancreatectomy hemorrhage; RBC, red blood cell; SSI, surgical site infection.

### Univariable and Multivariable Regression Analyses

Univariable and multivariable logistic regression analyses were performed on clinically relevant outcome variables, and the results are presented in Table 4. In the univariable regression analysis, DM was associated with the risk of SSI (odds ratio, 2.48 [95% CI, 1.04–5.96]; *P* = 0.04). After correcting for age, BMI estimated intraoperative blood loss and operating time. In a multivariable regression analysis, DM showed an independent relation with the risk of SSI, such that patients with DM had a 2.6-times-higher chance of developing an SSI (odds ratio, 2.60 [95% CI, 1.03–6.66]; *P* = 0.04) than those without. Effect modification by BMI on the chance of developing an SSI was not observed in the model (*P* > 0.92). No associations were found in both univariable and multivariable regression analyses for the remaining relevant outcome variables (ie, length of hospital stay ≥ 12 days, incidence of Clavien-Dindo complications ≥ 3b, and hospital readmissions). Finally, in univariable and multivariable logistic regression analyses, preoperative HbA_1c_ was not a predictor of the analyzed postoperative outcome variables.

**TABLE 4:** Univariable and Multivariable Logistic Regression Analyses on the Effect of DM on Postoperative Outcome Variables

Outcome	Predictor	β (95% CI)	Odds Ratio (95% CI)	*P*
Length of hospital stay ≥12 d	DM (ref: non-DM)	−0.39 (−1.28 to 0.47)	0.68 (0.28–1.60)	0.38
	DM (ref: non-DM)*	0.21 (−1.13 to 0.69)	0.81 (0.33–1.99)	0.65
SSI	DM (ref: non-DM)	0.91 (0.04–1.79)	2.48 (1.04–5.96)	**0.04**
	DM (ref: non-DM)^†^	0.98 (0.03–1.89)	2.68 (1.07–6.85)	**0.04**
Clavien-Dindo ≥ 3b complication	DM (ref: non-DM)	0.96 (−0.24 to 2.11)	2.61 (0.79–8.28)	0.10
	DM (ref: non-DM)*	0.95 (−0.32 to 2.20)	2.59 (0.72–9.03)	0.13
Hospital readmission	DM (ref: non-DM)	0.63 (−0.52 to 1.72)	1.88 (0.59–5.56)	0.26
	DM (ref: non-DM)*	0.68 (−0.49 to 1.80)	1.98 (0.61–6.06)	0.24

Significant values are indicated in bolded numbers.

β, beta; CI, confidence interval; DM, diabetes mellitus; SSI, surgical site infection.

*Adjusted for age, body mass index, and estimated intraoperative blood loss.

^†^Adjusted for age, body mass index, estimated intraoperative blood loss, and operating time.

### Subgroup Analysis

The subgroup analysis comparing patients with well-controlled (14 [48.2%]) and poorly controlled (15 [51.8%]) DM is provided in the supplemental materials. Other than the well-controlled DM group having more patients with pulmonary disease and the poorly controlled DM group having a higher prevalence of tobacco abuse, no differences were observed between the baseline patient characteristics of the 2 groups (Table S1, http://links.lww.com/MPA/B81). Furthermore, no significant differences in postoperative outcomes were observed between patients with adequately and poorly controlled DM (Table S2, http://links.lww.com/MPA/B82).

## DISCUSSION

In this study, the prevalence and effect of DM on postoperative outcomes in patients undergoing pancreatoduodenectomy were evaluated. Approximately one-quarter of the patients referred for pancreatoduodenectomy had DM, and 17% of these patients were given a diagnosis of new-onset DM. Notably, DM was considered poorly controlled in more than 50% of the patients with DM, with HbA_1c_ levels ≥ 7% (53 mmol/mol). In addition, DM was independently associated with a 2.6-times-higher risk of postoperative SSI in a multivariable regression analysis.

The results suggest that DM is a significant risk factor for SSI in pancreatic surgery. This finding is supported by multiple studies identifying DM as an independent risk factor for increased postoperative SSI rates in various surgical populations.^28–30^ However, although studies focusing on pancreatic surgery patients reported a high postoperative morbidity and readmission rate in patients with DM, they revealed no association between DM and SSI.^14,19^ The high rate of SSI in the DM group in this study may be attributable to the high percentage of patients with DM (>50%) with suboptimal glycemic control (HbA_1c_ > 7.0% [53 mmol/mol]) before surgery. Nevertheless, between patients with well-controlled and poorly controlled DM, no differences in the incidence of SSI were observed. Although the small sample size included in these analyses should be taken into account, it may suggest that postoperative glycemic control was suboptimal for all patients with DM in the cohort. Postoperative hyperglycemia, in both patients with and without DM, was directly associated with the risk of SSI.^31–33^ It has been suggested that pathophysiological alterations as a consequence of DM and hyperglycemia impair the immune system, increasing the risk of infectious complications.^34^ Hence, the World Health Organization guidelines recommend adequate perioperative glycemic control to reduce the risk of SSI in surgical patients.^35^ However, an effective tool for monitoring and improving perioperative glycemic control is currently lacking.

In this study, the preoperative HbA_1_c level was used to identify patients with new-onset DM and screen for poorly controlled DM. The measurement of HbA_1c_ is considered an adequate method for diagnosing DM and monitoring long-term glycemic control.^11,12^ Postoperative glucose control is dependent on point-of-care blood glucose measurement (POC-BGM). However, both HbA_1c_ and POC-BGM are point measurements that provide no information about (intraday and interday) glucose-level fluctuations. As such, HbA_1c_ and POC-BGM pose a risk of missing hypoglycemic and hyperglycemic episodes. Given the strong association between hypoglycemic and hyperglycemic episodes and DM-related surgical complications, this is a significant limitation of HbA_1c_ and POC-BGM.^36^ Consequently, hypoglycemic or hyperglycemic episodes could be missed.^37^ Continuous glucose monitoring (CGM) using a glucose sensor may be an effective way to optimize perioperative glycemic control in patients undergoing pancreatoduodenectomy by monitoring the patients' target glucose range.^36,38^ Continuous glucose monitoring monitors the glucose level in the interstitial fluid and provides a real-time warning if a trend toward hypoglycemia or hyperglycemia is detected. The median waiting time to surgery of 4 weeks provides an excellent opportunity to optimize preoperative glycemic control, particularly given the high prevalence of new-onset and poorly controlled DM. As such, CGM may be useful in achieving adequate glycemic control.^37^ Studies investigating the effect of CGM in pancreatic surgery reveal promising results, including improved perioperative glycemic control and fewer postoperative infectious complications.^38–40^

The strengths of this study include the use of HbA_1c_ and validated cutoff values from the European Association for the Study of Diabetes and the American Diabetes Association^11,13,41^ to identify new-onset and poorly controlled preoperative DM. Furthermore, independent associations between DM and postoperative outcomes were investigated by correcting for clinically relevant confounders. In addition to being screened for DM, the included patients were screened for other relevant patient-related risk factors in the new preoperative care pathway, in an effort to improve postoperative outcomes. Nevertheless, this study has limitations that need to be addressed. First, the HbA_1c_ cutoff point at ≥7% (53 mmol/mol) was used to differentiate between well- and poorly controlled DM. However, according to the American Diabetes Association guidelines, less stringent HbA_1c_ goals should be used when assessing glycemic control for certain patients (eg, older patients).^11^ In addition, although patients with poorly controlled DM before surgery were referred for glycemic control support, this intervention was not followed up on. Therefore, whether this intervention achieved the intended effect cannot be determined. However, because HbA_1c_ reflects the average plasma glucose level over the past 3 months and the median waiting time before surgery is 4 weeks, reevaluation of HbA_1c_ was considered not useful. Finally, the results of this study highlight the current lack of an effective measurement to monitor glycemic control in the preoperative and postoperative phases. This finding once again demonstrates the potential for CGM in optimizing adequate perioperative glycemic control, with implications for prehabilitation practice.

In conclusion, DM was diagnosed in approximately one-quarter of the patients scheduled for pancreatoduodenectomy. Diabetes mellitus poses a significant risk factor for patients scheduled for pancreatoduodenectomy, with high prevalence and rate of new-onset and poorly controlled DM. In addition, this study demonstrated that DM is independently associated with a high risk of developing a postoperative SSI. Adequate glycemic control is an essential component of perioperative care and has clinical implications for prehabilitation practice in pancreatic surgery.
